# Modulation of the Host Cell Transcriptome and Epigenome by Fusobacterium nucleatum

**DOI:** 10.1128/mBio.02062-21

**Published:** 2021-10-26

**Authors:** Cody A. Despins, Scott D. Brown, Avery V. Robinson, Andrew J. Mungall, Emma Allen-Vercoe, Robert A. Holt

**Affiliations:** a Canada’s Michael Smith Genome Sciences Centre, BC Cancer, Vancouver, British Columbia, Canada; b Department of Molecular Biology and Biochemistry, Simon Fraser University, Burnaby, British Columbia, Canada; c Molecular and Cellular Biology, University of Guelph, Guelph, Ontario, Canada; d Department of Medical Genetics, University of British Columbia, Vancouver, British Columbia, Canada; CIML

**Keywords:** *Fusobacterium nucleatum*, host response, transcriptome, epigenome, infection, oncomicrobe, colorectal cancer, *Fusobacterium*

## Abstract

Fusobacterium nucleatum is a ubiquitous opportunistic pathogen with an emerging role as an oncomicrobe in colorectal cancer and other cancer settings. F. nucleatum can adhere to and invade host cells in a manner that varies across F. nucleatum strains and host cell phenotypes. Here, we performed pairwise cocultures between three F. nucleatum strains and two immortalized primary host cell types (human colonic epithelial [HCE] cells and human carotid artery endothelial [HCAE] cells) followed by transcriptome sequencing (RNA-seq) and chromatin immunoprecipitation sequencing (ChIP-seq) to investigate transcriptional and epigenetic host cell responses. We observed that F. nucleatum-induced host cell transcriptional modulation involves strong upregulation of genes related to immune migration and inflammatory processes, such as *TNF*, *CXCL8*, *CXCL1*, and *CCL20*. Furthermore, we identified genes strongly upregulated in a cell line-specific manner. In HCE cells, overexpressed genes included *UBD* and *DUOX2*/*DUOXA2*, associated with p53 degradation-mediated proliferation and intestinal reactive oxygen species (ROS) production, respectively. In HCAE cells, overexpressed genes included *EFNA1* and *LIF*, two genes commonly upregulated in colorectal cancer and associated with poor patient outcomes, and *PTGS2* (*COX2*), a gene associated with the protective effect of aspirin in the colorectal cancer setting. Interestingly, we also observed downregulation of numerous histone modification genes upon F. nucleatum exposure. We used the ChIP-seq data to annotate chromatin states genome wide and found significant chromatin remodeling following F. nucleatum exposure in HCAE cells, with increased frequencies of active enhancer and low-signal/quiescent states. Thus, our results highlight increased inflammation and chemokine gene expression as conserved host cell responses to F. nucleatum exposure and extensive host cell epigenomic changes specific to host cell type.

## INTRODUCTION

Fusobacterium nucleatum is an elongated rod-shaped, anaerobic, Gram-negative bacterium found in both healthy and disease states of the human microbiota (1, 2). F. nucleatum is ubiquitous in the oral cavity and can spread beyond its oral niche through the bloodstream to colonize other body sites (3–6). For instance, F. nucleatum is an uncommon constituent of the gastrointestinal microbiota in healthy individuals (7, 8).

F. nucleatum colonization has been implicated in both oral and extraoral inflammatory human diseases such as periodontitis (9, 10), endodontic infection (11, 12), inflammatory bowel disease (13), appendicitis (14), and adverse pregnancy outcomes (15, 16). Moreover, F. nucleatum is an emerging oncomicrobe; it is enriched in colorectal cancer (17, 18), breast cancer (19), esophageal cancer (20), oral/head and neck cancers (21), and melanoma (22). F. nucleatum may have a role in tumorigenesis and/or metastatic spread given that it is associated with precancerous colonic polyps (23, 24) and metastatic disease (25–27). Clinically, an increased F. nucleatum tumor burden is associated with poor patient outcomes (28), chemoresistance (29), and relapse (30). Thus, there is a strong rationale to investigate F. nucleatum pathogenicity.

It is well established that F. nucleatum can adhere to host cells and invade the host cytosol; however, these characteristics vary markedly among F. nucleatum strains and host cell types (13, 31, 32). The virulence mechanisms of F. nucleatum and how they may relate to host cell adhesion or invasion are not well understood. The FadA adhesin is a candidate F. nucleatum virulence factor, shown to bind E-cadherin and activate Wnt/β-catenin signaling, a pathway frequently dysregulated in colorectal cancers (33, 34). F. nucleatum has also been linked to increased DNA damage (35, 36), possibly related to nuclear localization after invasion (32, 37). Furthermore, the invasiveness of F. nucleatum strains isolated from inflammatory bowel disease patients correlates with disease severity (13), but it is not known if this is generalizable to other disease settings.

The interaction between F. nucleatum and host immunity is similarly complex, with F. nucleatum displaying inflammatory and immunogenic properties but also having immunosuppressive effects. For example, F. nucleatum induces proinflammatory cytokines (24, 33, 38, 39) and can stimulate local myeloid cell recruitment (24). Additionally, F. nucleatum can induce adaptive immune responses; colorectal cancer patients have increased levels of anti-F. nucleatum antibodies compared to healthy controls (40, 41), and anti-F. nucleatum CD8^+^ T cells can infiltrate F. nucleatum-positive melanoma tumors (22). However, F. nucleatum can also act in an immunosuppressive manner. F. nucleatum outer membrane proteins can induce apoptosis in lymphocytes (42, 43), and F. nucleatum tumor burden is inversely correlated with CD3^+^ T cell density in colorectal cancer (44). Furthermore, F. nucleatum inhibits NK and T cell responses via fusobacterial lectin Fap2 binding to the inhibitory TIGIT and CEACAM1 host receptors (45, 46), and F. nucleatum can also stimulate tryptophan metabolism to yield kynurenine metabolites that are potent inhibitors of T cell function (47, 48). Overall, the host-pathogen interactions of F. nucleatum are multifactorial, and the consequences of F. nucleatum tumor enrichment and invasion, such as the potential for oncogenic initiation and/or progression, are not fully understood.

Recently, we investigated transcriptional changes in F. nucleatum upon host cell invasion and found modulation of genes that may contribute to hematogenous spread and the generation of a tumor-permissive hypoxic/inflammatory microenvironment (4). In the present study, we explore the effects of F. nucleatum on the host cell transcriptome and epigenome. We exposed human immortalized primary (IP) colonic epithelial (HCE) and vascular endothelial (human carotid artery endothelial [HCAE]) cells to three strains of F. nucleatum *in vitro* using previously established coculture conditions permissive to F. nucleatum adhesion and invasion (4, 13) and then performed transcriptome sequencing (RNA-seq) and chromatin immunoprecipitation sequencing (ChIP-seq) on F. nucleatum-exposed and -unexposed host cell populations. We found that under different conditions, F. nucleatum exposure upregulated genes related to inflammation (chemokines and cytokines), downregulated genes related to histone modification, and significantly remodeled chromatin states.

## RESULTS

### Exposure to F. nucleatum results in upregulation of host inflammatory responses and downregulation of host histone modification-related genes.

To investigate the host cell response to F. nucleatum strains in different human cell types, we performed coculture assays in triplicate using two immortalized primary (IP) cell lines (human colonic epithelial [HCE] and human carotid artery endothelial [HCAE] cells) exposed to three different strains of F. nucleatum (F. nucleatum subsp. *animalis* 7/1 [F. nucleatum 7/1] [13], F. nucleatum subsp. *animalis* CC 7/3 JVN3C1 [F. nucleatum 7/3] [4], and F. nucleatum subsp. *nucleatum* ATCC 23726 [F. nucleatum ATCC 23726]). These colonic epithelial and vascular endothelial host cell lines were chosen to explore the consequences of F. nucleatum exposure/invasion relevant to colorectal cancer pathogenesis and the capacity of F. nucleatum to transit from the bloodstream to other tissues, respectively. After 4 h of F. nucleatum exposure to host cells, we extracted host cell RNA for transcriptome analysis (see Materials and Methods). We performed differential gene expression analysis independently for each combination of F. nucleatum strain and host cell, identifying differentially expressed (DE) transcripts that are upregulated or downregulated in F. nucleatum-exposed samples relative to unexposed control cells processed in parallel. For our analysis, we focused on protein-coding genes. Of the 60,671 total annotated human transcripts from Ensembl (49), 19,966 are classified as protein coding.

**FIG 1:**
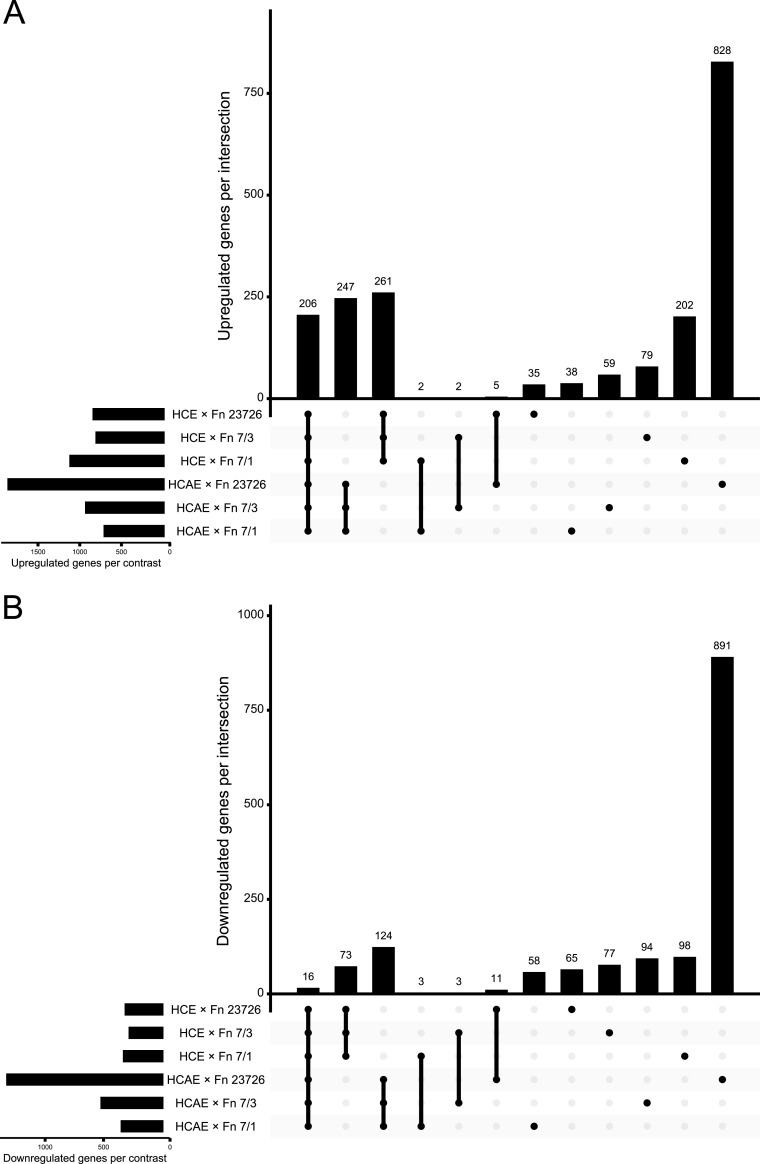
Comparison of HCE and HCAE cell protein-coding genes differentially expressed in response to various F. nucleatum (Fn) strains. Upset plots highlight the number of shared and unique significantly differentially expressed upregulated (A) and downregulated (B) genes. Horizontal bars at the bottom left of each plot show the total number of significantly differentially expressed genes under each condition. Vertical bars show the number of unique or shared genes between each comparison, with conditions included in each comparison shown by the respective filled points below.

We first sought to determine the similarity of transcriptional changes under each condition of host cell and F. nucleatum strain. We found a large set of significantly DE genes that were shared under all conditions (206 upregulated and 16 downregulated) (Fig. 1A; see also Table S1 in the supplemental material). We also found hundreds of genes consistently modulated within the same cell line exposed to different F. nucleatum strains (F. nucleatum strain-nonspecific, host cell line-specific changes), while few genes were consistently modulated between the two different cell lines exposed to the same F. nucleatum strain (F. nucleatum strain-specific, host cell line-nonspecific changes). This suggests that modulation of the host cell transcriptome by F. nucleatum is mainly defined by host cell identity rather than F. nucleatum strain identity. Interestingly, however, the HCAE cell line showed an exceptionally large number of uniquely DE genes when exposed to F. nucleatum ATCC 23726 (828 upregulated and 891 downregulated, compared to 35 to 202 upregulated and 58 to 98 downregulated unique genes under other conditions), indicating the potential for context-specific host-pathogen interactions.

10.1128/mBio.02062-21.2 TABLE S1 Shared and unique significantly differentially expressed genes for each host cell and F. nucleatum strain condition. Gene names and shared or unique condition assignments for significantly (adjusted *P* value of ≤0.05) differentially expressed upregulated and downregulated genes are shown (related to Fig. 1). Download Table S1, XLS file, 0.1 MB. Copyright © 2021 Despins et al. 2021 Despins et al. https://creativecommons.org/licenses/by/4.0/ This content is distributed under the terms of the Creative Commons Attribution 4.0 International license.

**FIG 2:**
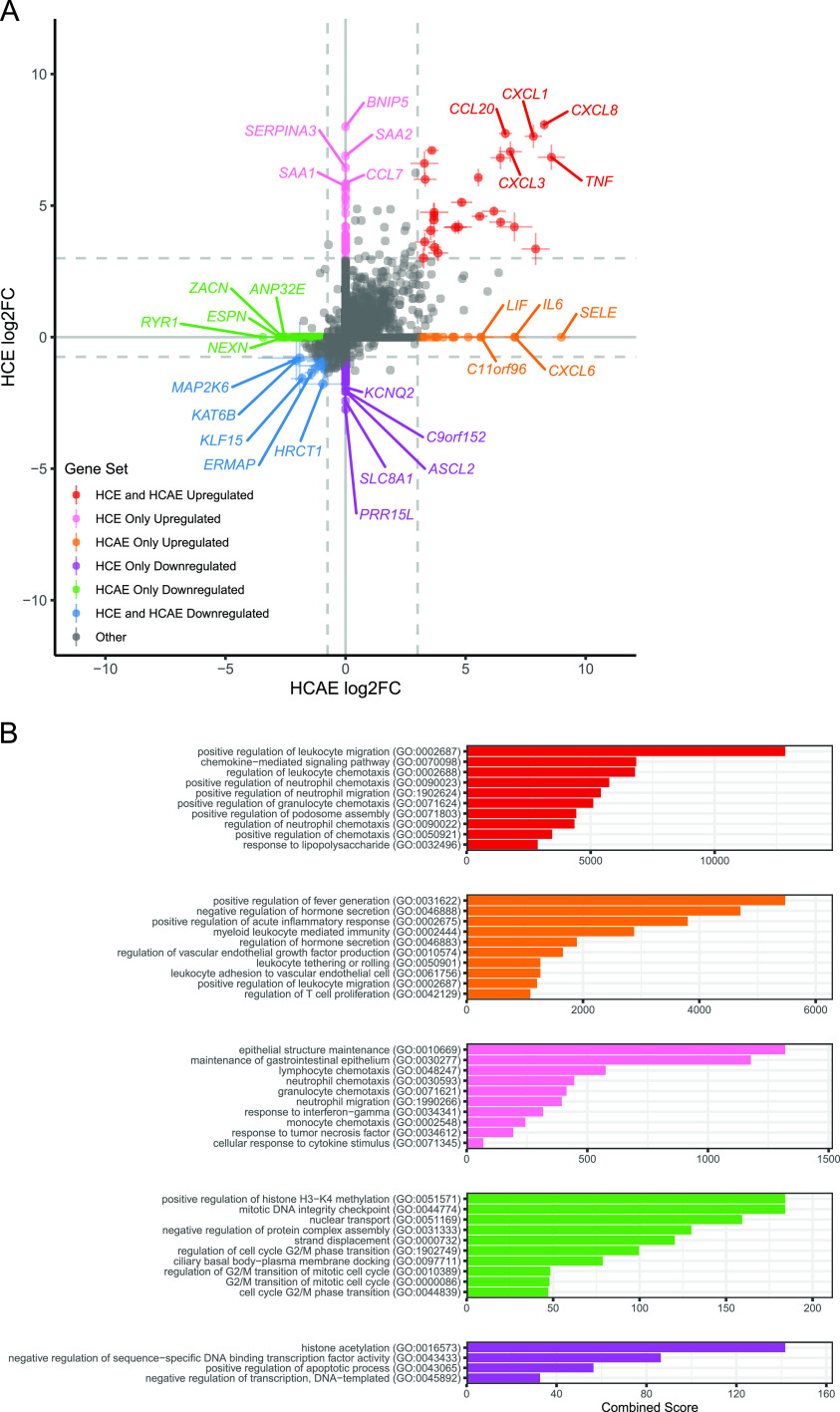
Differential expression gene sets of interest and related pathways. (A) Scatterplot and gene set assignments of DE genes for HCE and HCAE cells. For all genes that were significantly DE (adjusted *P* ≤ 0.05) under at least 1 condition, the mean and standard deviation of DE genes for each IP cell exposed to three different F. nucleatum strains were calculated (nonsignificant DE gene log2FC = 0) (crossbars denote standard deviations). Genes with an average log2FC of greater than 3 (upregulated) or less than −0.75 (downregulated) were considered genes of interest (colored points) (the top 5 genes of each group are labeled). Genes meeting these criteria in both cell lines were considered to be conserved upregulated or downregulated genes of interest. Genes meeting these criteria in one cell line and not significantly DE in the other cell line were considered cell line-specific genes of interest. (B) Gene set enrichment analysis (GSEA) of gene sets of interest. Ensembl gene names of each gene set were used separately as inputs for EnrichR GSEA, querying the GO biological process library. Significant resulting GO terms (adjusted *P* ≤ 0.05) were ranked by combined score (up to 10 terms shown). No significant GO terms were identified for the HCE and HCAE downregulated (blue) gene set.

We next aimed to identify the strongest transcriptional changes in response to F. nucleatum exposure in both host cell lines. The mean and standard deviation of each DE gene for each IP cell line exposed to three different F. nucleatum strains were calculated (nonsignificant DE gene log_2_ fold changes [log2FCs] = 0). Genes with an average log2FC of greater than 3 (for upregulated genes) or less than −0.75 (for downregulated genes) under both conditions were considered conserved genes of interest (Fig. 2A, red and blue points, with the top 5 genes of each group labeled). Using these criteria, we identified 26 genes that were consistently upregulated between conditions of cell lines and F. nucleatum strains (*BIRC3*, *CCL2*, *CCL20*, *CSF2*, *CSF3*, *CXCL1*, *CXCL10*, *CXCL2*, *CXCL3*, *CXCL5*, *CXCL8*, *ELF3*, *ICAM1*, *KCNN3*, *LTB*, *NFKBIA*, *OLR1*, *OR2I1P*, *RHCG*, *RND1*, *RRAD*, *TNF*, *TNFAIP2*, *TNFAIP3*, *TNFRSF9*, and *ZC3H12A*). We also identified 17 genes that were consistently downregulated between conditions of cell lines and F. nucleatum strains (*AP5S1*, *ARFGAP2*, *ASNS*, *BMI1*, *ERMAP*, *FANCE*, *HRCT1*, *ID2*, *KAT6B*, *KLF15*, *LPIN2*, *MAP2K6*, *MXD3*, *SH3TC2*, *TTC30B*, *ZBTB3*, and *ZNF367*). These are the host genes showing the largest and most consistent expression changes upon F. nucleatum exposure.

Next, we identified the strongest transcriptional changes in response to F. nucleatum exposure that were unique to each host cell line. We considered genes that met our minimum DE cutoffs (average log2FC of greater than 3 or less than −0.75) in one cell line and were not significantly differentially expressed in the other cell line as cell line-specific genes of interest (Fig. 2A, orange, purple, pink, and green points, with the top 5 genes of each group labeled; Table S2). The identified “HCE-only” (106 genes) and “HCAE-only” (270 genes) gene sets represent the transcriptional changes specific to each host cell context, and these genes may therefore be important for F. nucleatum pathogenesis related to colorectal cancer/inflammatory bowel disease and hematogenous spread, respectively.

10.1128/mBio.02062-21.3 TABLE S2 Top differentially expressed gene sets of interest. Gene names, log2FC values under each condition, means and standard deviations for each cell line, and gene set assignments for genes significantly differentially expressed under at least 1 condition (nonsignificant DE gene log2FC = 0) are shown (related to Fig. 2). Download Table S2, XLS file, 0.1 MB. Copyright © 2021 Despins et al. 2021 Despins et al. https://creativecommons.org/licenses/by/4.0/ This content is distributed under the terms of the Creative Commons Attribution 4.0 International license.

To explore cellular processes associated with the differentially expressed gene sets of interest, gene set enrichment analysis (GSEA) was performed using EnrichR (50). The “GO biological process 2018” ontology was queried, and significantly enriched terms were ranked by combined score (Fig. 2B). We observed a number of chemokine- and immune cell migration-related terms associated with both our conserved upregulated gene set (Fig. 2B, red) and our cell line-specific upregulated gene sets (orange and pink) (“positive regulation of leukocyte migration,” “chemokine-mediated signaling pathway,” and multiple terms related to the chemotaxis of leukocytes/neutrophils/monocytes/granulocytes). Additionally, many terms were related more generally to proinflammatory responses (“positive regulation of acute inflammatory response,” “positive regulation of fever generation,” “response to lipopolysaccharide,” “response to interferon gamma,” “response to tumor necrosis factor,” and “cellular response to cytokine stimulus”). This finding suggests that the induction of inflammatory and immune recruitment genes dominates the host cell responses to F. nucleatum exposure. Interestingly, we also observed terms specific to each of the host cell lines. For example, using genes DE only in HCE cells as the input, the two most strongly associated GO terms relate to maintenance of the gastrointestinal epithelium (“epithelial structure maintenance” and “maintenance of gastrointestinal epithelium”). Alternatively, we also noted processes specific to HCAE cells that align with vascular cell-specific functions (“regulation of vascular endothelial growth factor production,” “leukocyte tethering or rolling,” and “leukocyte adhesion to vascular endothelial cell”). For GSEA of downregulated gene sets of interest, no significant GO terms were identified for the conserved downregulated gene set (genes seen in Fig. 2A, blue points). For cell line-specific downregulated gene sets, we found that both HCE and HCAE cell gene lists generated top GO terms related to histone modification (“histone acetylation” and “positive regulation of histone H3-K4 methylation,” respectively). In addition, a number of terms related to cell cycle progression processes were associated with downregulated genes in HCAE cells (“mitotic DNA integrity checkpoint” and multiple terms relating to the regulation of the G_2_/M-phase transition).

**FIG 3:**
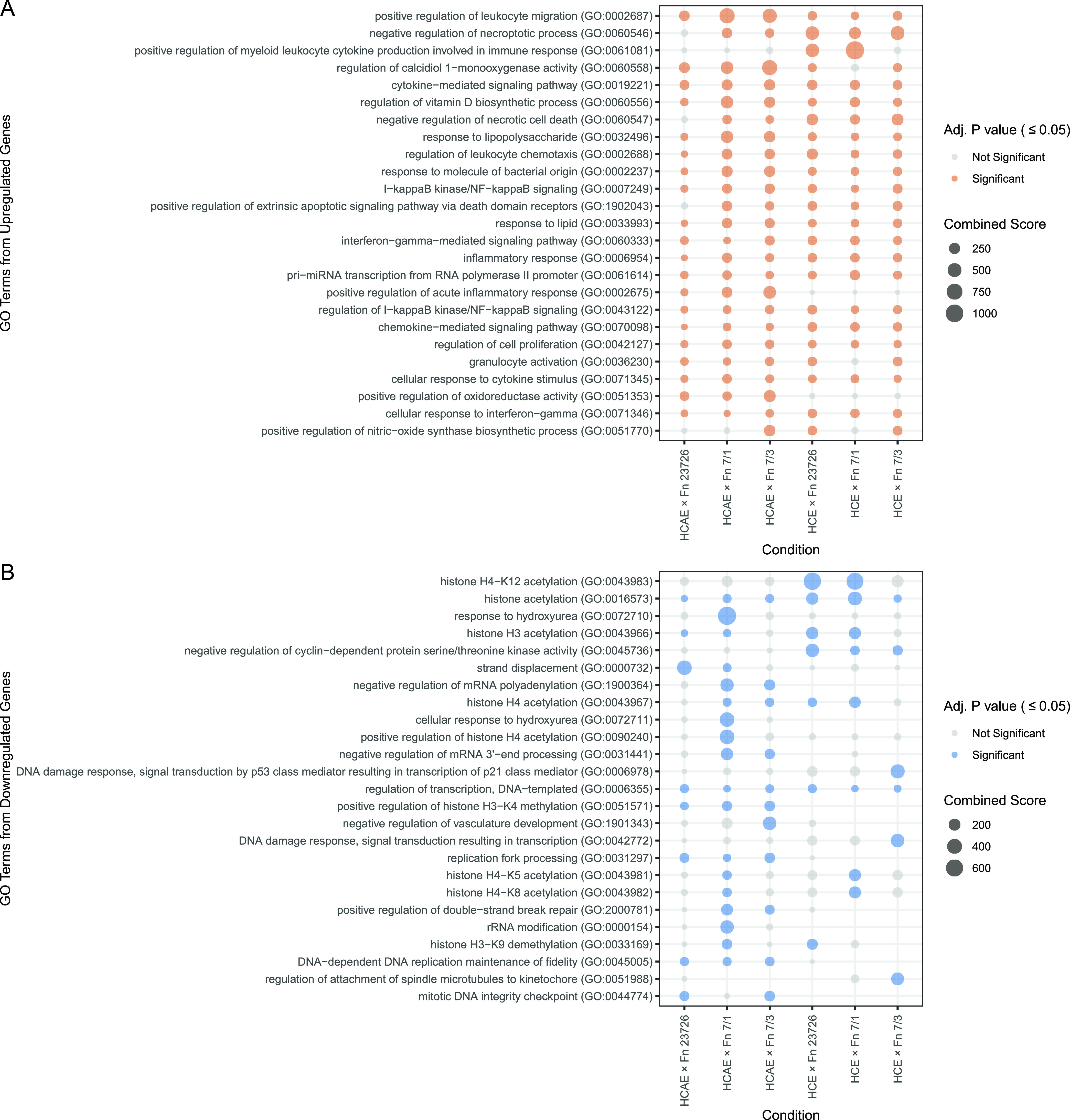
Gene set enrichment analysis (GSEA) of all significantly differentially expressed human protein-coding genes in response to various F. nucleatum (Fn) strains. The *y* axis shows the top 25 GO terms for upregulated (A) and downregulated (B) gene sets for all conditions (ranked by the sum of the “total score” of significant GO terms from all conditions from top [highest] to bottom [lowest]). Significant GO term total scores (adjusted *P* value of ≤0.05) are colored (orange, upregulated; blue, downregulated), and nonsignificant GO term total scores are shown in gray. The size of the dot reflects the score of the GO term for each condition.

We performed GSEA including all significantly upregulated and downregulated genes separately for each combination of host cell and F. nucleatum strain to see if any reoccurring terms emerged that may have been unrepresented in the top gene set analysis. We ranked the significant output GO terms by the sum of their combined scores for each cell line and F. nucleatum strain to compare enrichment across all conditions. Similar to trends seen when looking at the strongly upregulated genes of the HCE and HCAE cell upregulated set, enriched GO terms from the complete set of upregulated genes included terms associated with inflammation and immune cell migration (Fig. 3A). Emphasizing the importance of results from the conserved downregulated gene subset, a clear trend of histone modification term enrichment emerged, particularly those terms related to acetylation, when considering all downregulated genes (“histone acetylation” [generally as well as specific to H3, H4, H4-K5, H4-K8, and H4-K12], “positive regulation of histone H3-K4 methylation,” and “histone H3-K9 demethylation”) (Fig. 3B). In this more inclusive analysis, we also observed NF-κB signaling as a previously unidentified top upregulated process (“IκB kinase/NF-κB signaling” and “regulation of IκB kinase/NF-κB signaling”). Additionally, DNA damage-related processes were associated with downregulated genes (“DNA damage response, signal transduction by p53 class mediator resulting in transcription of p21 class mediator” and “DNA damage response, signal transduction resulting in transcription”).

### ChIP-seq reveals epigenetic signatures altered by F. nucleatum-induced epigenetic changes.

**FIG 4:**
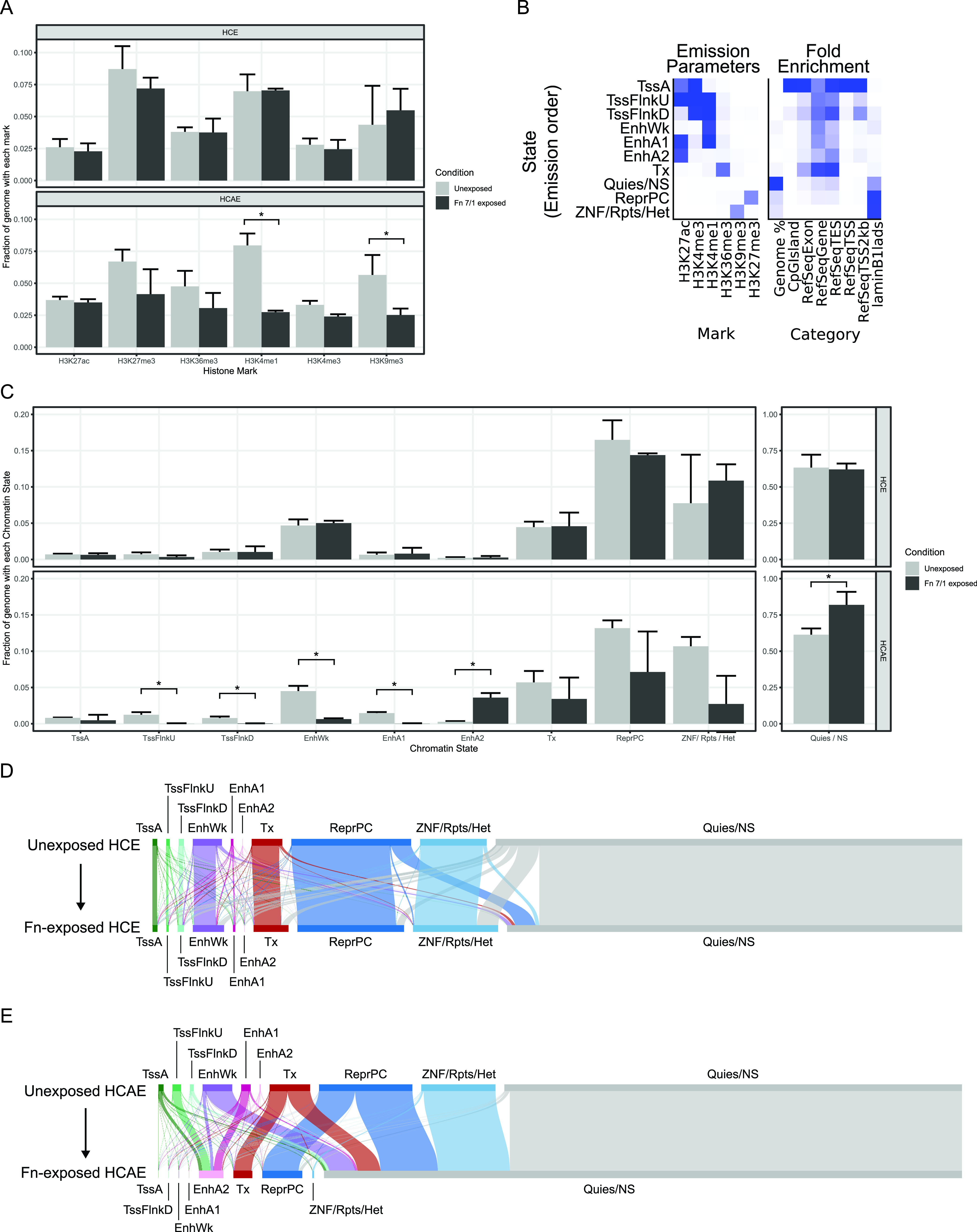
Epigenetic profiling of HCE and HCAE cells exposed to F. nucleatum (Fn) 7/1. (A) Bar plots showing the fraction of the genome that contains each histone mark (*x* axis) in control (light gray) and F. nucleatum 7/1-exposed (dark gray) HCE (top) and HCAE (bottom) cells. The bar height is the mean from three replicates, with the error bar extending to the mean and the standard deviation. (B) Summary of the 10-state ChromHMM model emission parameters (left) and genomic annotation enrichments (right). The intensity of blue correlates with increasing values of the emission parameter or fold enrichment. (C) Bar plots showing the fraction of the entire genome that is classified as each state (*x* axis) in control (light gray) and F. nucleatum 7/1-exposed (dark gray) HCE (top) and HCAE (bottom) cells. The bar height is the mean from the three replicates, with the error bar extending to the mean and the standard deviation. The Quies/NS state is shown in its own panel with a different *y* axis scale. (D and E) Sankey diagrams showing the changes in states for every window in the unexposed (top) and F. nucleatum 7/1-exposed (bottom) HCE (D) and HCAE (E) cells. Bar sizes for each state are proportional to the amount of the genome represented by that state. Totals of 96.9% (HCE cells) and 96.5% (HCAE cells) of the total genome length are represented in these diagrams. Connections from unexposed state (top) to exposed state (bottom) are colored by the unexposed state.

Prompted by the observed downregulation of histone methylation/acetylation pathways in the differential gene expression analysis, we evaluated histone modifications in response to F. nucleatum. For these experiments, we exposed HCE and HCAE cells to the F. nucleatum 7/1 strain as this well-characterized strain is highly invasive and has been shown to promote tumorigenesis in mice (24). We performed ChIP-seq experiments for the six core histone marks used by the International Human Epigenome Consortium (IHEC) (H3K4me1, H3K4me3, H3K9me3, H3K27me3, H3K27ac, and H3K36me3) (51). After alignment of the sequencing data to the human genome, we divided the genome into nonoverlapping 200-bp windows (*n* = 15,478,375) using ChromHMM (52), which used the aligned ChIP-seq data to assign the absence or presence of each mark in each of the windows. Next, the overall fractions of the genome containing each mark were compared (Fig. 4A). In HCAE cells, there was a significant decrease in the fraction of the genome containing H3K4me1 (*P* = 0.0095 by a *t* test) and a smaller decrease in H3K4me3 (*P* = 0.019). In HCE cells, there were no significant changes in the fractions of the genome containing any of the six marks. These observations suggest that F. nucleatum may influence the epigenome in a cell type-specific manner.

To further explore epigenomic modulation by F. nucleatum, we used ChromHMM to create a model of chromatin states. Chromatin states are defined by the combination of histone marks co-occurring in regions of the genome (53–55). We selected a model with 10 chromatin states based on published chromatin state models (56, 57) and manual inspection of the emission parameters and genomic characteristics of each state (Fig. 4B). Our 10 chromatin states can be divided into five groups: promoter (active TSS [transcriptional start site] [TssA], flanking active TSS upstream [TssFlnkU], and flanking active TSS downstream [TssFlnkD]), enhancers (weak enhancer [EnhWk], active enhancer 1 [EnhA1], and active enhancer 2 [EnhA2]), genic transcription (transcription [Tx]), repressed (repressed polycomb [ReprPC] and ZNF genes, repeats, and heterochromatin [ZNF/Rpts/Het]), and low signal (quiescent/no signal [Quies/NS]). Using this model, we assigned every 200-bp window of the genome its best-evidenced chromatin state. When looking at the fraction of the genome that was classified as being in each state, we observed that for both HCE and HCAE cells, the majority of the genome was classified as the Quies/NS state (Fig. 4C).

Changes in the genome-wide abundance of chromatin states in response to F. nucleatum exposure were strikingly different in HCAE cells compared to HCE cells. In HCAE cells, we saw significant decreases in TssFlnkU, TssFlnkD, EnhWk, and EnhA1 and increases in EnhA2 and Quies/NS (*P* < 0.05). The largest changes in HCAE cells were in the “Quies/NS” state (20.6% increase) and “EnhWk” (3.9% decrease). In HCE cells, there were no significant changes in any of the states. We summarized the specific state changes undergone by each window of the genome in a Sankey diagram (Fig. 4D and E). This view highlights the predominant state transitions in HCAE cells upon F. nucleatum exposure, including substantial shifts from the TssFlnkU and EnhA1 states to the EnhA2 state and from the EnhWk, Tx, ReprPC, and ZNF/Rpts/Het states to the Quies/NS state.

### F. nucleatum-associated chromatin state changes correlate with F. nucleatum-associated gene expression changes.

Given that we found regions of the HCAE genome with changes in the chromatin state upon exposure to F. nucleatum, we sought to correlate the specific state changes with changes in gene expression. We identified 4,456,888 windows in the HCAE genome that changed upon F. nucleatum exposure. To ascribe the epigenetic impact of these windows to specific genes, we used GREAT (58) to associate each genomic region with one or more genes using the basal regulatory region rules (see Materials and Methods). To assign a quantitative value to the cumulative effect on transcription for genes with associated state changes, we scored each state change based on the predicted effect of the state on gene expression (+2, TssA and Tx; +1, TssFlnkU, TssFlnkD, EnhWk, EnhA1, and EnhA2; 0, Quies/NS; −1, ReprPC and ZNF/Rpts/Het) and determined the difference in values between the ending state and the starting state to assign an “epigenomic score” value to each window. Next, we summed all epigenomic scores for all windows associated with a specific gene to obtain a net epigenomic score for each gene. Focusing on each set of genes identified in Fig. 2A, we measured the correlation between the log2FC and the net epigenomic score for these genes and tested for significance by a random resampling method (see Materials and Methods). We saw significant correlations for HCAE upregulated genes (HCE and HCAE upregulated [Fig. 2A, red points], *r* = 0.559 and *P* = 0.02; HCAE-only upregulated [orange points], *r* = 0.506 and *P* = 0.03) and downregulated genes (HCE and HCAE downregulated [Fig. 2A, blue points], *r* = 0.517 and *P* = 0.04). Repeating this analysis for HCE cells, we saw no significant correlations between expression changes and epigenomic changes, further substantiating the sensitivity of the HCAE cell line to epigenomic modulation by F. nucleatum.

### F. nucleatum invades IP vascular endothelial cells and IP colonic epithelial cells.

To investigate the possible role of invasion in our RNA-seq and ChIP-seq data, we repeated coculture assays for all combinations of IP cell line and F. nucleatum strain. After 4 h of F. nucleatum exposure to host cells, invasion was assessed by microscopy using 4′,6-diamidino-2-phenylindole (DAPI) (staining host cell nuclei and F. nucleatum nucleoids) and anti-F. nucleatum polyclonal antibodies (Fig. S1). We found evidence of invasion (F. nucleatum with DAPI-stained nucleoids but lacking membrane staining) in HCAE and HCE cells by all 3 F. nucleatum strains. Qualitatively, we observed that HCAE cells were more susceptible to invasion than HCE cells. F. nucleatum invasion may therefore contribute to the differential expression and epigenetic changes observed in our data, particularly for HCAE cells.

10.1128/mBio.02062-21.1 FIG S1 Representative fluorescence microscopy images of each host cell and F. nucleatum (Fn) strain condition. HCAE (top) and HCE (bottom) host cell lines were stained with DAPI, primary rat anti-F. nucleatum antibodies, and secondary goat anti-rat IgG antibodies conjugated to cyanine 3 (Cy3). DAPI (blue) stains host cell nuclei as well as F. nucleatum cell nuclei of intracellular and extracellular F. nucleatum. Cy3 F. nucleatum staining (red) labels only extracellular F. nucleatum. F. nucleatum nuclei appear as punctate blue points along the elongated F. nucleatum body. White arrows highlight invading F. nucleatum cells, characterized by blue points near the host cell nuclei without any red staining. Download FIG S1, PDF file, 1.7 MB. Copyright © 2021 Despins et al. 2021 Despins et al. https://creativecommons.org/licenses/by/4.0/ This content is distributed under the terms of the Creative Commons Attribution 4.0 International license.

## DISCUSSION

Fusobacterium nucleatum is among the most common bacterial species of the oral cavity and is becoming increasingly recognized as an opportunistic pathogen, implicated in diseases including numerous cancer types and both oral and extraoral infections. Despite such inculpatory findings, the understanding of F. nucleatum pathogenesis and virulence factors that contribute to disease development is complicated by F. nucleatum strain diversity and differential effects on host cells and ultimately remains limited.

Here, we have comprehensively profiled transcriptional and epigenetic host cell changes in response to F. nucleatum exposure. To address possible differences in host cell responses between F. nucleatum strains and host cell types, we performed pairwise coculture assays for 2 different human immortalized primary cell lines using 3 separate F. nucleatum strains.

Our results indicate that F. nucleatum exposure upregulates genes related to immune migration and inflammatory processes, consistent with previous reports. For example, F. nucleatum treatment increased the recruitment of tumor-associated macrophages and dendritic cells (DCs) and induced the expression of inflammatory genes in the Apc*^min^*^/+^ mouse model (24). The human colorectal cancer-derived HCT116 cell line has been shown to upregulate *CXCL8* (*IL-8*) and *CXCL1* in response to F. nucleatum ATCC 23726 and may contribute to metastatic spread (59). Modulation of these genes is corroborated by our data, with *CXCL8* and *CXCL1* being among the most highly upregulated genes in both the HCE and HCAE cell lines upon exposure to all F. nucleatum strains. *TNF* has also been reported to be induced by multiple invasive F. nucleatum strains in LS 174T colonic cells (60). We show that this proinflammatory cytokine is upregulated strongly by both HCE and HCAE cells. *CCL20* recruits regulatory T (Treg) cells and is associated with chemoresistance in the setting of colorectal cancer (61). Upregulation of *CCL20* and *CSF-3* (*G-CSF*) has been shown in F. nucleatum-infected esophageal tumor tissue and gingival fibroblasts, respectively (20, 62). *CSF-2* (*GM-CSF*) upregulation has been observed in F. nucleatum-infected gingiva-derived mesenchymal stem cells (63), and a role for *CSF-2* in the colorectal cancer epithelial-to-mesenchymal transition has been proposed (64). We show that the upregulation of *CCL20*, *CSF-3*, and *CSF-2* is induced in both HCE and HCAE cells upon exposure to all F. nucleatum strains tested in our study. These collective findings therefore suggest that the upregulation of these genes may be an important feature of F. nucleatum-induced host responses consistent between various host cell settings and F. nucleatum strains. Previous work has shown a negative correlation between F. nucleatum load and CD3^+^ T cell density (44). A proposed mechanism for the decrease of CD3^+^ T cell density related to F. nucleatum load is through F. nucleatum-induced recruitment of myeloid-derived suppressor cells (MDSCs), which can inhibit the proliferation of T cells and induce apoptosis in T cells. Indeed, MDSCs have been found to be enriched by F. nucleatum in mouse models (24). Numerous immune recruitment-related genes differentially expressed in response to F. nucleatum in our data have been shown to lead to MDSC infiltration in some cancer settings, such as *CCL20*/*CCL2* (65), *CXCL5* (66), and *CXCL1* (67). Therefore, our data support the recruitment of MDSCs that could contribute to the decreased CD3^+^ T cell content associated with F. nucleatum load (44). Overall, our results extend the emerging trend of inflammatory and immune migration-related gene upregulation as a conserved key aspect of the host response to F. nucleatum exposure.

Recent evidence suggests a role of F. nucleatum in promoting chemoresistance in colorectal cancer patients (29). Furthermore, upregulation of *BIRC3*, an inhibitor of apoptosis, has been implicated as a primary mediator of this effect (68). *BIRC3* was strongly upregulated upon F. nucleatum exposure under every coculture condition of our study, suggesting that the upregulation of this gene and related effects on chemoresistance may be conserved between various F. nucleatum strains and host cell types.

Gene set enrichment analysis (GSEA) also revealed several notable pathways modulated upon F. nucleatum exposure. F. nucleatum has been previously shown to increase host cells in S phase and G_2_/M phase (69, 70). We show that G_2_/M-phase cell cycle-related terms are among the most enriched biological process GO terms associated with the HCAE cell-specific downregulated gene set. The genes related to these terms (*AKAP9*, *CDK5RAP2*, *CEP57*, *CEP135*, *CEP290*, *CNTRL*, *KIF14*, *NINL*, *PPP1R12A*, and *USP47*) therefore may contribute to changes in host cell proliferation and tumor size following exposure to F. nucleatum, as has been previously reported (70). Additionally, we observed upregulation of genes related to gastrointestinal maintenance in HCE cells (*MUC2* and *SERPINA3*), possibly relevant to disruption of gastrointestinal barrier integrity by F. nucleatum (71).

Our analysis also highlights genes that may be differentially expressed in response to F. nucleatum in a host cell-specific manner. *UBD* upregulation is correlated with increased tumor size, more advanced disease stages, increased recurrence, and decreased survival in colorectal cancer patients, possibly via the degradation of p53 and the promotion of cell proliferation (72, 73). In our data, HCE cells significantly upregulated *UBD* in response to F. nucleatum exposure. *DUOX2* is upregulated in inflammatory bowel disease and colorectal cancers and, alongside *DUOXA2*, is a primary driver of H_2_O_2_ production in mucosal tissues, which may contribute to chronic inflammation and reactive oxygen species (ROS)-related DNA damage (74, 75). *DUOX2* and *DUOXA2* were both strongly upregulated by HCE cells in response to F. nucleatum, suggesting that F. nucleatum may drive H_2_O_2_ production in the setting of the colon. *PTGS2* (*COX2*) upregulation is associated with F. nucleatum abundance in colon tumors (24). Additionally, aspirin, a modulator of *PTGS2*, has long been recognized for its chemopreventive effect in the setting of colorectal cancers (76) and was recently demonstrated to reduce the development of colonic adenomas after F. nucleatum inoculation in the Apc*^min^*^/+^ mouse model (77). In our data, *PTGS2* is among the most strongly differentially expressed genes in HCAE cells but is not differentially expressed in HCE cells, suggesting that the upregulation of *PTGS2* and aspirin inhibition may be particularly important in some host cell or tissue contexts. Additionally, we found that *EFNA1* (ephrin A1) and *LIF* (leukemia-inhibitory factor) are also differentially expressed only in HCAE cells. The overexpression of both *EFNA1* and *LIF* is common in colorectal cancer and associated with poor patient outcomes, and *LIF* expression mediates chemoresistance via the negative regulation of p53 *in vitro* (78, 79). Thus, these genes and others observed to be modulated only in specific host cells in this study represent cell type-specific candidates that can be evaluated for their relationship to pathogenesis and correlation with disease severity in future studies.

Previously, Hong et al. (80) reported histone acetylation of specific genes following F. nucleatum-induced long noncoding RNA (lncRNA) ENO1-IT upregulation. To our knowledge, our present study is the first to explore epigenome-wide changes in host chromatin in response to F. nucleatum exposure. Strikingly, we saw significant epigenomic changes in HCAE cells but not in HCE cells, suggesting that these changes may be linked to host cell type. Furthermore, we showed that the level of differential gene expression in highly differentially expressed genes correlates with these chromatin changes, supporting F. nucleatum-induced epigenomic restructuring and transcriptomic dysregulation.

To assess which of our coculture conditions showed evidence of host cell invasion by F. nucleatum, we stained cells with DAPI and anti-F. nucleatum polyclonal antibodies and used microscopy to visualize our cocultures. Evidence of the invadability of both host cell lines by each F. nucleatum strain was observed (see Fig. S1 in the supplemental material), suggesting that some of the transcriptional and epigenetic host cell responses to F. nucleatum could be driven by F. nucleatum gaining access to the host cytosol. Qualitatively, HCAE cells were more invadable by F. nucleatum than HCE cells, and therefore, some of the prominent changes noted in HCAE cells, including the striking changes in chromatin states, may be related to host cell invasion. Future studies deploying single-cell analytical methods capable of discriminating between invaded and uninvaded host cells will be needed to verify transcriptomic and epigenetic changes associated with invasion.

We recognize further limitations to our experimental design. Our study investigated host cell responses to F. nucleatum in human colonic epithelial cells and human carotid artery endothelial cells with one immortalized primary cell line representing each cell type. The transcriptional and epigenetic changes associated with these cell lines may or may not reflect the responses of colonic epithelial cells and vascular endothelial cells generally. Future studies using additional cell lines from a variety of tissue sources will be needed to further refine cell-of-origin effects.

In summary, we have identified candidate genes and gene pathways that are dysregulated in host cells cocultured with F. nucleatum. Additionally, we have shown genome-wide and specific chromatin state changes associated with HCAE cells, which correlate with observed changes in gene expression. Our results highlight upregulation of inflammation and chemokine gene expression and downregulation of histone modification-related genes as common host cell responses to F. nucleatum exposure and demonstrate epigenetics as a new area of research toward defining F. nucleatum pathogenicity.

## MATERIALS AND METHODS

### Immortalized primary cell maintenance and preparation for coincubation.

Two immortalized primary (IP) cell lines were obtained from suppliers: human carotid artery endothelial (HCAE) cells (catalog number T0512; ABM) and human colonic epithelial (HCE) cells (catalog number T0570; ABM). HCAE and HCE cells were maintained in PriGrow I and III (ABM), respectively, supplemented with 10% fetal bovine serum (FBS) and 50 μg/ml Plasmocin. IP cells were allowed to reach 100% confluence before washing with 0.2-μm-filter-sterilized phosphate-buffered saline (PBS) and harvested using 0.25% trypsin-EDTA (Thermo Fisher) for 5 min at 37°C with 5% (vol/vol) CO_2_. Following trypsin quenching by the addition of the respective cell media, IP cells were collected by centrifugation at 300 × *g* for 3 min and seeded into a new flask at a 1:5 seeding ratio. Once IP cells reached 70% confluence at passages 2 to 7, the medium was aspirated, and the cells were washed gently in PBS. Serum- and antibiotic-free Dulbecco’s modified Eagle’s medium (DMEM) was then applied in preparation for coincubation.

### F. nucleatum strain maintenance and preparation for coincubation.

Three F. nucleatum strains were used in this study: F. nucleatum subsp. *animalis* 7/1 (F. nucleatum 7/1) (also reported as EAVG_002) isolated from an inflammatory bowel disease patient (13), F. nucleatum subsp. *animalis* CC 7/3 JVN3C1 (F. nucleatum 7/3) isolated from a colorectal cancer patient (4), and F. nucleatum subsp. *nucleatum* ATCC 23726 (F. nucleatum ATCC 23726). F. nucleatum strains were maintained in a humidified anaerobic chamber (Ruskinn BugBox) at 37°C in 10% (vol/vol) CO_2_, 1% (vol/vol) H_2_, and balanced N_2_ on fastidious anaerobe agar (FAA; Neogen) supplemented with 5% (vol/vol) defibrinated sheep’s blood (Hemostat Laboratories). F. nucleatum broth cultures for coincubations were grown overnight (16 to 18 h) to late log phase in tryptic soy broth (TSB) supplemented with 5 μg/ml hemin and 1 μg/ml menadione, 0.2-μm-filter sterilized (supplemented TSB), that had been previously incubated anaerobically for >16 h (degassed). The F. nucleatum cultures were then diluted with degassed supplemented TSB to 10^8^ CFU ml^−1^, verified by MacFarland standards.

### Coincubation of immortalized primary cells and F. nucleatum.

Coincubations between F. nucleatum and human cells were completed in biological triplicate at separate human cell passages. Diluted F. nucleatum broth cultures were applied to IP cells at a multiplicity of infection (MOI) of 50:1 fusobacterial cells-host cells. IP cells were incubated with F. nucleatum 7/1, F. nucleatum 7/3, or F. nucleatum ATCC 23726 or alone (control) for 4 h at 37°C with 5% (vol/vol) CO_2_.

### Cell harvest for RNA extraction.

Following a 4-h coincubation, cells were gently washed twice with PBS. Cells were harvested using 0.25% trypsin-EDTA (Thermo Fisher) for 5 min at 37°C with 5% (vol/vol) CO_2_, neutralized with the respective media containing 10% (vol/vol) FBS, and collected by centrifugation at 300 × *g* for 3 min. Cells were then resuspended in 1 ml TRIzol (Thermo Fisher), incubated for 3 min at room temperature, and then frozen at −20°C until RNA harvest. RNA was extracted from the TRIzol samples according to the manufacturer’s standards. Briefly, samples were allowed to come to room temperature and then combined with 0.2 ml chloroform. Samples were incubated for 2 min prior to centrifugation at 12,000 × *g* for 15 min at 4°C. The aqueous phase was carefully removed by pipetting and combined with 0.5 ml isopropanol. The mixture was incubated for 10 min and then centrifuged at 12,000 × *g* for 10 min at 4°C. The supernatant was carefully discarded by pipetting. The RNA pellet was then washed twice in 75% ethanol, collected by centrifugation at 7,500 × *g* for 5 min at 4°C, and then air dried for 10 min. The RNA pellet was then resuspended in 50 μl nuclease-free water and incubated for 10 min at 55°C. The RNA was then treated with dsDNase (Thermo Fisher) according to the manufacturer’s standards. The quantity and quality of the RNA were assessed by Agilent TapeStation 4150 analysis. All samples submitted for RNA sequencing had RNA integrity number (RIN) values of >7.5.

### Cell harvest for chromatin immunoprecipitation.

Following a 4-h coincubation of F. nucleatum 7/1 with each of the IP cell lines or IP cells alone, cells were gently washed twice with PBS and harvested using 0.25% trypsin-EDTA at 37°C with 5% (vol/vol) CO_2_. Trypsin was then neutralized with the respective media containing 10% (vol/vol) FBS, and cells were collected by centrifugation at 300 × *g* for 3 min. Cells were then washed once in PBS and harvested by centrifugation at 300 × *g* for 3 min. PBS was then aspirated, and cells were immediately snap-frozen in liquid N_2_. Frozen cell samples were stored at −80°C prior to analysis.

### Chromatin extraction and immunoprecipitation.

The generation of ChIP-seq libraries from frozen cell pellets was performed by Canada’s Michael Smith Genome Sciences Centre, Vancouver, Canada. For each sample, immunoprecipitation was performed for six core histone marks (H3K9me3, H3K27me3, H3K36me3, H3K4me1, H3K4me3, and H3K27ac). ChIP libraries were made for each of these marks, and one was made for the input control DNA, yielding seven libraries per sample. Libraries were constructed with IDT’s duplex unique molecular identifiers (UMIs) for improved duplicate read detection.

### RNA sequencing.

RNA sequencing was performed by Canada’s Michael Smith Genome Sciences Centre, Vancouver, Canada. For each sample, ribosomal RNA-depleted strand-specific RNA-seq libraries were constructed. All sequencing was performed using 150-bp paired-end reads on an Illumina HiSeq 2500 instrument. Each of the 24 libraries was individually barcoded, pooled, and sequenced at approximately 10 libraries per lane. The results of sequencing are summarized in Table S3 in the supplemental material.

10.1128/mBio.02062-21.4 TABLE S3 Summary of RNA-seq and ChIP-seq data. Total numbers of reads and reads aligned for each replicate under each condition from RNA-seq are shown. Total numbers of reads and reads aligned for each replicate of each histone mark under F. nucleatum-unexposed and F. nucleatum-exposed conditions from ChIP-seq are indicated. Download Table S3, XLS file, 0.03 MB. Copyright © 2021 Despins et al. 2021 Despins et al. https://creativecommons.org/licenses/by/4.0/ This content is distributed under the terms of the Creative Commons Attribution 4.0 International license.

### ChIP sequencing.

ChIP sequencing was performed by Canada’s Michael Smith Genome Sciences Centre, Vancouver, Canada. Libraries were sequenced using 150-bp paired-end reads on an Illumina HiSeqX instrument at a target depth of 100 million reads for H3K9me3, H3K27me3, H3K36me3, H3K4me1, and input control DNA, while H3K4me3 and H3K27ac had a target depth of 50 million reads. The results of sequencing are summarized in Table S3.

### RNA-seq data processing.

RSEM (81) rsem-calculate-expression was used to align raw RNA-seq data sets to the GRCh38 reference genome and calculate gene expression values (ensembl101) (49). Differential gene expression was assessed using DESeq2 (82), using the established workflow for processing RSEM-generated data [setting the minimum gene length to 1 for all genes after running tximport() and before running DESeqDataSetFromTximport()]. This workflow includes estimation of size factors, estimation of dispersion, and negative binomial generalized linear model (GLM) fitting. Log fold change shrinkage was performed using the normal prior.

### RNA-seq data analysis.

Differentially expressed genes were selected by an adjusted *P* value threshold of 0.05. Ensembl identifiers were mapped to gene names using ensembl101. To define top gene sets of interest associated with various conditions, the mean and standard deviation of DE genes for each IP cell exposed to three different F. nucleatum strains were calculated for all genes that were significantly DE (adjusted *P* ≤ 0.05) under at least 1 condition (nonsignificant DE gene log2FC = 0). Genes with an average log2FC of greater than 3 (upregulated) or less than −0.75 (downregulated) were considered genes of interest for the respective conditions, as shown in Fig. 2A.

### GSEA of differentially expressed genes.

The top gene sets of interest shown in Fig. 2A, as well as all significantly differentially expressed genes (selected by an adjusted *P* value threshold of 0.05), had Ensembl identifiers mapped to gene names using ensembl101. These gene names were used as the input for EnrichR. In Fig. 2B, GO terms were ranked by the “total score” of significant GO terms from top (highest) to bottom (lowest). In Fig. 3, GO terms were ranked by the sum of the total score of significant GO terms from all conditions from top (highest) to bottom (lowest).

### ChIP-seq data processing.

UMIs were extracted, and sequences were mapped to the human reference genome hg19 via BWA-MEM. Deduplication was performed with picard tools MarkDuplicates. ChromHMM was used to binarize the aligned ChIP-seq data for each replicate individually. In the ChromHMM design file, the input DNA ChIP-seq data were provided as a local control for each mark to control for local variances in genome coverage. Chromatin state models containing 2 to 24 states were learned using the LearnModel command, and all models were compared to the Roadmap Epigenomics Consortium (REC) (56) 18-state model, which was defined using the same six histone marks that we investigated. By assessing the median correlation of the 18 states in each of our potential models, we found 10 states to be the smallest number of states that maximized the correlation. By correlating the emission parameters of each state in our 10-state model to the emission parameters of the REC 18-state model and by manual inspection of the genomic annotations, we classified our states based on the REC 18-state model.

### ChIP-seq data analysis.

To obtain a higher-confidence set of chromatin state annotations for our samples, we identified the regions that had consistent states called across each of the three replicates. Here, we defined a consistent state as at least two of the three replicates with the same state assignment, and we scanned through the genome to identify each consistent 200-bp window. Windows that did not have matching states across at least two of the three replicates in both the unexposed and F. nucleatum-exposed samples were categorized as not having a consistent state and were removed from further analysis. Having a single state call for every 200-bp window for each sample, we then compared all regions in the control and F. nucleatum-exposed samples to identify the state change associated with each region that correlates with F. nucleatum exposure.

### Integration of transcriptomic and epigenomic data.

To integrate the chromatin state changes with transcriptomic changes, we first used GREAT (58) to find gene associations for these regions using the default “basal plus extension” association rule modified to not allow distal associations (0 kb) and not including curated regulatory domains. We then defined a net epigenomic score for each gene, which quantifies the summative effect of all associated windows on the gene. States were assigned scores based on the predicted effect on transcription (+2, TssA and Tx; +1, TssFlnkU, TssFlnkD, EnhWk, EnhA1, and EnhA2; 0, Quies/NS; −1, ReprPC and ZNF/Rpts/Het), and the score for each window was calculated as the score for the state in the F. nucleatum-exposed sample minus the score for the state in the unexposed sample. The net epigenomic score was calculated for each gene by taking the sum of scores for all windows associated with that gene. To test the correlation with gene expression changes, we calculated the Pearson correlation coefficient between the gene expression value (log2FC) and the net epigenomic score for subsets of genes identified in the gene expression analysis. To test the statistical significance of these correlations, we randomly sampled equally sized sets of genes and tested the correlation for these random subsets, repeated this 1,000 times, and recorded the *P* value as the fraction of these 1,000 trials that resulted in a correlation coefficient more extreme (farther from zero) than the observed correlation coefficient.

### Immunofluorescence visualization of F. nucleatum and immortalized primary cell coincubations.

IP cells were grown to 70% confluence at passage 2 on coverslips pretreated with poly-l-lysine (Sigma). Briefly, coverslips were incubated for 5 min in 1 mg/ml poly-l-lysine at room temperature and then washed once with sterile deionized water. Coverslips were allowed to dry for >2 h at room temperature before seeding with IP cells at a 1:5 seeding ratio. Coincubations of each F. nucleatum strain and IP cell line were completed in biological triplicate. Following a 4-h coincubation, cells were gently washed twice with PBS. Cells were then fixed with 4% paraformaldehyde (Sigma) for 15 min at room temperature. Paraformaldehyde was then aspirated, and cells were blocked for >24 h with 10% normal goat serum (NGS) (Thermo Fisher) at 4°C. External F. nucleatum cells were stained for 30 min at 37°C with primary rat anti-F. nucleatum antibodies: F. nucleatum 7/1 with EAV_AS4 (diluted 1/500 in NGS), F. nucleatum 7/3 with EAV_AS2 (diluted 1/200 in NGS), and F. nucleatum ATCC 23726 with EAV_AS7 (diluted 1/500 in NGS) (13). EAV_AS7 was prepared alongside previously reported rat antibody sera and has been determined to be reactive with F. nucleatum ATCC 23726 by immunofluorescence microscopy (data not shown). Cells were then washed vigorously for 5 s three times with PBS. Goat anti-rat IgG antibodies conjugated to cyanine 3 (Cy3) (Thermo Fisher) diluted 1/500 in NGS containing 1 μg/ml DAPI were then applied to the cells. Cells were allowed to incubate for 30 min at room temperature, in the dark. Cells were then washed vigorously for 5 s three times with PBS. Coverslips were mounted onto microscopy slides by applying Mowiol 4-88 (Sigma) and allowed to solidify overnight (14 to 20 h), in the dark. Fluorescence micrographs of stained IP cells and F. nucleatum cells were collected on a Leica DM 5000B instrument using Semrock-DAPI and Texas Red filters.

### Data availability.

The RNA-seq and ChIP-seq data generated in this study have been submitted to the NCBI BioProject database under accession number PRJNA730807. Custom code used in this study is available in the GitHub repository at https://github.com/scottdbrown/host-transcriptome-epigenome-fuso.
