# Supplementary material for: Modulation of the Host Cell Transcriptome and Epigenome by Fusobacterium nucleatum
Source: mBio. 2021 Oct 26;12(5):e02062-21. doi: 10.1128/mBio.02062-21 (PMC8546542; doi:10.1128/mBio.02062-21)
Supplement: FIG S1 [file mbio.02062-21-sf001.pdf]

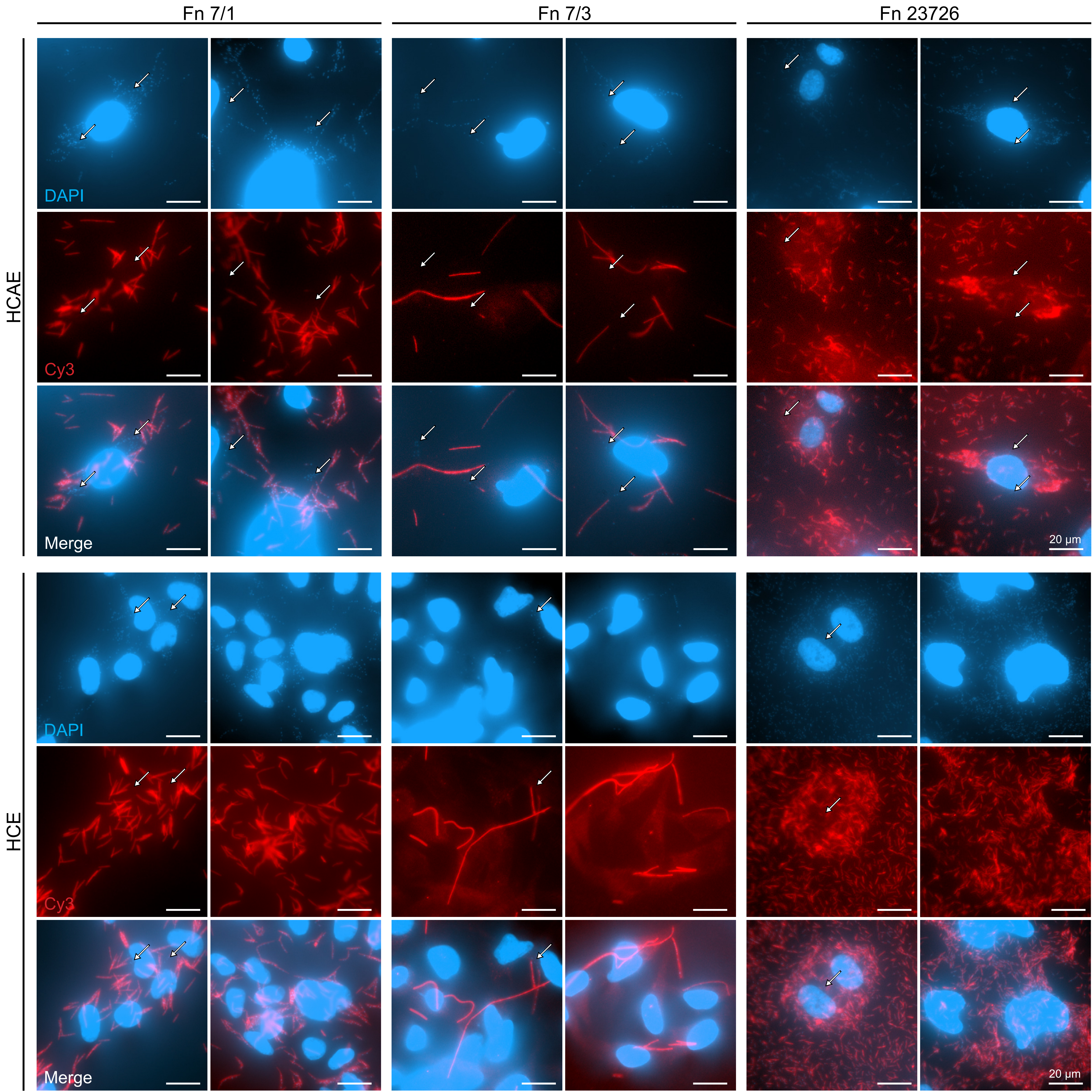

Fn 7/1
Fn 7/3
Fn 23726
HCAE
HCE
DAPI
Cy3
Merge
20 µm
